# Synthesis and Biological Evaluation of New 5-Fluorouracil-Substituted Ampelopsin Derivatives

**DOI:** 10.3390/molecules15042114

**Published:** 2010-03-24

**Authors:** Wei-Ming Zhou, Rong-Rong He, Jian-Tao Ye, Na Zhang, De-Yu Liu

**Affiliations:** 1 School of Pharmaceutical Sciences, Sun Yat-sen University, Guangzhou, 510006, China; E-Mails: weiming81@126.com (W.-M.Z.); herongrong22@163.com (R.-R.H.); yejt@mail.sysu.edu.cn (J.-T.Y.); nanon120@yahoo.com.cn (N.Z.); 2 Guangdong Food and Drug Vocational College, Guangzhou 510520, China;

**Keywords:** ampelopsin, 5-fluorouracil, anticancer activity

## Abstract

This study reports two novel 5-fluorouracil-substituted ampelopsin derivatives. The structures of two new derivatives were characterized by elemental analysis, ^1^H-NMR, ^13^C-NMR, IR and MS. Their anticancer activities *in vitro* against two cancer cell lines, K562 and K562/ADR, were investigated using the MTT assay, and the results showed that the two new compounds were more effective than reference drugs such as ampelopsin and verapamil.

## 1. Introduction

Ampelopsin (3,5,7,3′,4′,5′-hexahydroxyl-2,3-dihydroflavonol, CAS: 27200-12-0, **1**) is a purified component of the tender stems and leaves or roots of the Chinese medicinal herbs *Ampelopsis cantoniensis *(Hook.et Arn) Planch or *Ampelopsis grossedentata *(Hand. Mazz.) W.T. Wang (Vitaceae) [1]. It is also known as ampeloptin or dihydromyricetin. In traditional Chinese herbal medicine, ampelopsin has detoxication and anti-inflammation functions. Our previous studies have proved that ampelopsin additionally possesses powerful antineoplastic and antioxidant properties [2,3]. It can induce the apoptosis of cancer cells and inhibit the growth of tumor vessels by depressing the expression of VEGF and *b*FGF [4]. However, the chemical constitution of ampelopsin is not stable. In aqueous or organic solution, ampelopsin decomposed under exposure to light, and a colored photolysis product was formed [5]. Several studies have been carried out to improve the stability of ampelopsin by modifying its chemical structure [6,7,8]. Thus, the stability may also be improved through the combinations of another anticancer agent and ampelopsin that give rise to change chemical structure. These combinations which offer new possibilities to treat solid malignancies may provide the basis for considering effective combination cancer chemotherapy as protective and symptomatic treatment. 

5-Fluorouracil (5-Fu, CAS: 51-21-8) (**2**) is a good candidate for cancer treatment, especially for gastrointestinal tumors [9,10,11,12,13], which was first synthesized in 1957 [14]. It is one of the most frequently used antitumor agents for the treatment of solid tumors, such as breast, colorectal, and gastric cancers [15]. However, clinical use is limited by its serious side effects [16]. The various methods attempting to reduce its side effects have included synthesis of 5-Fu derivatives with low molecular weight [17], conjugation with other compounds [18], entrapment in polymer devices [19] and attachment to polymer chains [20]. These *N*-1 and/or *N*-3 substituted derivatives have exhibited improved pharmacological and pharmacokinetic properties, including increased bioactivity, selectivity, metabolic stability, absorption and lower toxicity [21].

**Figure 1:**
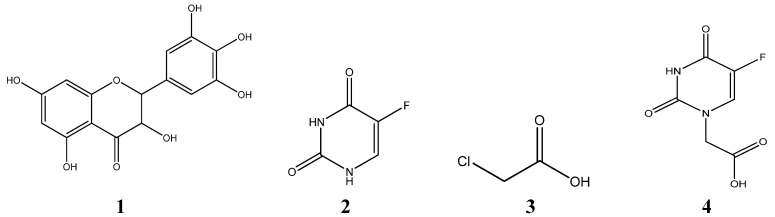
Structures of the reactants.

**Scheme 1:**
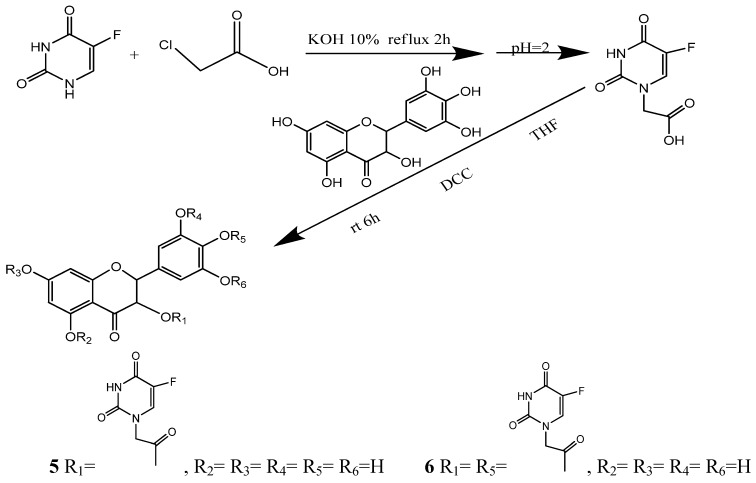
Synthesis and structures of compounds **5, 6**.

Herein, we report that the synthesis of two novel 5-Fu-substituted ampelopsin derivatives with bimolecular structures joined by chloroacetic acid (**3**) (Scheme 1). The solvent used in this study was tetrahydrofuran (THF) and the condensing agent was *N,N′*-dicyclohexylcarbodiimide (DCC) [22]. The derivatives were characterized by elemental analysis, ^1^H-NMR, ^13^C-NMR, IR and MS. In addition, the *in vitro* anticancer activity of these new compounds was evaluated by a MTT assay in the K562 cancer cell line, and their effects in reversing multidrug resistance was determined in K562/ADR cells.

## 2. Results and Discussion

### 2.1. Synthesis and Post-Treatment

For the synthesis of 5-fluorouracil-1-carboxylic acid (**4**), the mixture of 5-Fu and potassium hydroxide was refluxed for 2 h in 70 °C after adding chloroacetic acid, then cooled to room temperature and acidified to pH 5.5 with concentrated hydrochloric acid. After cooling at 4 °C for 2 h, the resulting crystals were isolated by suction and the solution was harvested, and the unreacted 5-Fu was eliminated [23]. Under these conditions, the reaction product existed in the solution as organic anion. The solution was further acidified to pH 2 with concentrated hydrochloric acid and cooled at 4 °C for another 6 h. This treatment promoted complete crystallization that raises the yield of 5-fluorouracil-1-carboxylic acid.

To optimize the conditions for the ester condensation reaction between 5-Fu and ampelopsin, different catalysts, solvents, the reaction time and ratio of the reactants were investigated. The results revealed that conventional catalysts including 4-dimethylaminopyridine (DMAP) and pyridine did not influence the yield of **5**. Among the three solvents investigated, acetone and THF were better than acetic ester in which the reactants had the worst solubility. Since acetone is a controlled substance in China, THF was used as the reaction solvent. The results also showed that the yield of **5 ** reached its peak at 6 h and did not increase even after extending the reaction time. *N*-(3-dimethylaminopropyl)-*N*-ethylcarbodiimidehydrochloride (EDC·HCl), *N,N′-*dicyclohexylcarbodiimide (DCC) and N,N′-diisopropylcarbodiimide (DIC) [24,25,26] were used in this study as condensing agents, respectively. The results proved that DCC preferable because it could increase the solubility of reactants which in turn raised the production rate. In addition, the products varied with the ratio of reactants used. When the ratio between **1** and **3** was less than 1:1.2, only **5** was obtained. However, both **5 **and **6** could be detected when the ratio was more than 1:1.2. Moreover, the yields of **5** and **6** increased along with the proportion of **3 **in the reactants. When the ratio increased to 1:4, the yields reached its peak which was 62.3% for **5** and 28.6% for **6**, respectively.

### 2.2. Chemical Characterization

**Figure 2:**
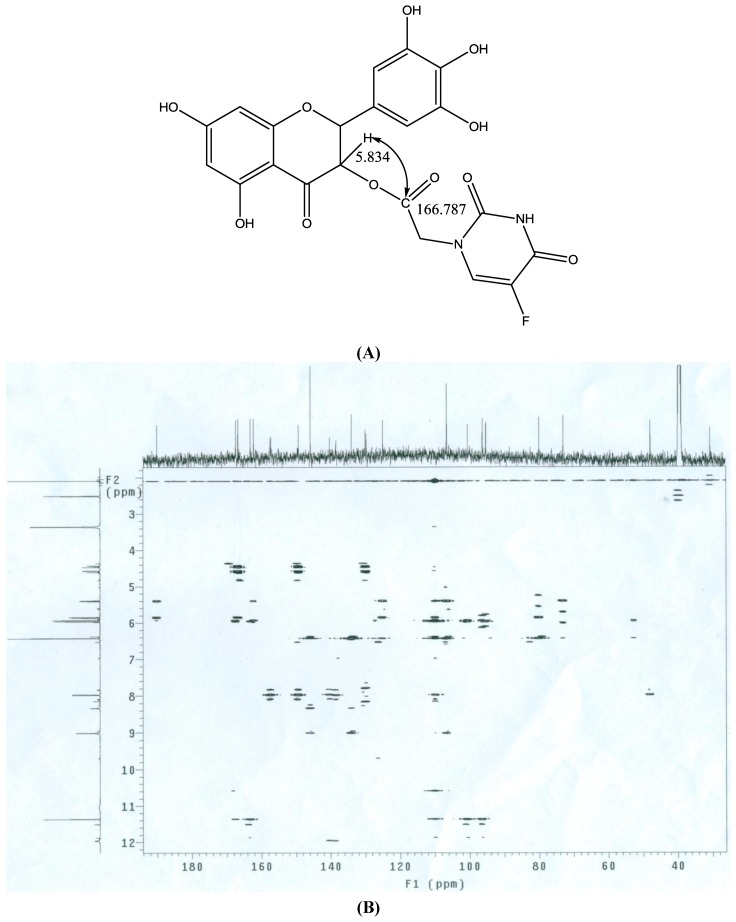
gHMBC of **5**.

The structure of compound **5** was established by spectroscopic (MS, IR, ^1^H-NMR, ^13^C-NMR) as well as elemental analysis data. The MS showed peaks at 489.0 (M-H) and 978.8 (2M-H) with negative ion, meanwhile peaks at 513.1 (M + Na), 1018.9 (2M + K) and 1508.6 (3M + K) were detected in positive ion mode. Thus, the MS data are in agreement with the values calculated for the expected molecular formula. The IR spectra displayed two characteristic NH group bands at 3,112 cm^−1^ and 1,458 cm^−1^, respectively, and the C=O group band which is absent in compound **1** appeared at 1,756 cm^−1^. In the ^1^H-NMR spectra the NH groups in the pyrimidine heterocycle were identified at 5.971 ppm and 11.950 ppm while the peak for the C_3-_OH in **1** simultaneously disappeared. The C-O signal appeared at 162.28 ppm in the ^13^C-NMR. Based on the 2D-NMR spectra (gCOSY, gHMQC and gHMBC), it was proven that the reaction took place at C_3_-OH by gHMBC (Figure 2), since the extension level line and the extension vertical line separately intersected with peak 5.834 ppm in ^1^H-NMR and 166.787 ppm in ^13^C-NMR, indicating C-H group at C_3_ of the flavonoid had a relationship with the acetyl group in 5-fluorouracil-1-carboxylic acid (**4**).

The structure of compound **6** was characterized by spectroscopic (IR, ^1^H-NMR, ^13^C-NMR) as well as elemental analysis data. The IR spectra showed that the two NH group bands at 3,078 cm^−1^ and 1,472 cm^−1^. The signals of the three hydroxyl groups in **6** (C_3_-OH, C_7_-OH and C_4’_-OH) could not be detected in the ^1^H-NMR spectra. According to the structure of **5** described before, it was known that the condensation reaction took place first at C_3_-OH. In the ^1^H-NMR spectrum the signal of C_7_-OH disappeared but turned up after heated. In addition the signals of C_3’_-OH and C_5’_-OH groups in **6 **was observed at 9.769 ppm which were different from that observed at 9.003 ppm in **5**. Hence, it was believed that the condensation reactions occurred in C_3_-OH and C_4’_-OH positions. Thermal gravimetric analysis showed that **5** existed as a trihydrate, while **6** is a tetrahydrate. The crystallization water in **5** and **6**may come from the environment during storage.

### 2.3. Biological Activity

The anticancer activities of these new derivatives were tested by the MTT assay in leukemia cell lines K562 and K562/ADR [28]. The cells were treated with **5** and **6 **for 24, 48 and 72 h, and the resulting IC_50_ values are shown in Table 1. The inhibitory effects of **5** and **6** reached a peak at 48 h, and did not increase further with extended incubation time. In K562 cells, there was no significant difference between the IC_50_ values of **5**, **6**, and their physical mixtures with 5-Fu, suggesting that the new derivatives did not have additive effects when co-administrated with 5-Fu. However, in K562/ADR cells, **5** was more effective than AMP and the mixtures, which indicated the potential anti-cancer effect of these new compounds in drug resistant cancer cells. In addition, the IC_50_ of ADR in K562 and K562/ADR cells were 1.26 ± 0.15 μmol/L and 50.52 ± 4.03 μmol/L respectively, which showed that K562/ADR cells were markedly resistant to ADR.

We further investigated the reversal effects of **5** and **6 **against drug resistance in K562/ADR cells. In this experiment, the IC_50_ of ADR was detected at the present of verapamil, **5**, **6**, AMP, 5-Fu and AMP plus 5-Fu, respectively (Table 2). The concentrations of 0.625 μmol/L and 1.25 μmol/L were chosen, because all the compounds showed no significant cytotoxicity when used alone at these concentrations (inhibition rate ≤10%). The results showed that both **5** and verapamil could significantly reverse the drug resistance of K562/ADR cells, and they were more effective than the physical mixture of AMP and 5-Fu. At the concentration of 0.625 μmol/L, the reversal effect of **5** was even better than that of verapamil. **6** also had a weak effect at the concentration of 1.25 μmol/L. The reversal effect may be contributed to the increased sensitivity of cancer cells to ADR at the present of low concentration of novel compounds. Although the mechanism underlying remains to be clarified, our findings indicated the potential use of 5-Fu-substituted AMP derivatives in the drug resistance of cancer. 

**Table 1:** The inhibitory effects of **5 **and **6** on K562 and K562/ADR cells.

Group	K562			K562/ADR 48 h IC_50 _ (μmol/L)
	24 h IC_50 _ (μmol/L)	48 h IC_50 _ (μmol/L)	72 h IC_50 _ (μmol/L)	
AMP	20.55 ± 1.10	13.78 ± 2.14	10.25 ± 0.33	38.89 ± 4.77
**5**	22.50 ± 2.65	11.62 ± 2.20	12.54 ± 1.02	7.19 ± 0.51
**6**	21.51 ± 1.84	10.34 ± 0.60	11.11 ± 1.48	11.26 ± 1.29
AMP+5-Fu /1:1	--	11.29 ± 0.56	--	32.63 ± 2.67
AMP+5-Fu /1:2	--	12.68 ± 1.33	--	29.20 ± 2.29
ADR	--	1.26 ± 0.15	--	50.52 ± 4.03

IC_50_ values were the mean ± S.D. of six independent experiments.

**Table 2:** Reversal effect of **5** and **6** on multidrug resistancein K562/ADR cells for 48 h.

Group	Concentration (μmol/L)	IC_50 _(μmol/L)	Fold-reversal
Control	--	50.52 ± 4.03	1.00
Verapamil	0.625	20.72 ± 1.62 **	2.44
	1.25	12.00 ± 1.26 **	4.21
**5**	0.625	17.94 ± 1.09 **	2.82
	1.25	14.45 ± 1.52 **	3.50
**6**	0.625	48.88 ± 8.30	1.03
	1.25	36.16 ± 4.49 **	1.40
AMP	0.625	38.34 ± 4.20 *	1.32
	1.25	20.19 ± 4.29 **	2.50
5-Fu	0.625	47.03 ± 2.79	1.07
	1.25	41.12 ± 2.13	1.23
AMP+5-Fu /1:1	0.625	39.78 ± 2.84 *	1.27
	1.25	27.04 ± 4.51 **	1.87

IC_50_ values were the mean ± S.D. of six independent experiments. * *P *< 0.05, ** *P *< 0.01 *vs*. control group (treated with ADR only). Fold-reversal = IC_50_ (control group)/IC_50_ (drug-treated group).

## 3. Experimental

### 3.1. General

IR spectra were taken as KBr disks on a Bruker EQUINOX 55 FTIR Spectrometer. ^1^H-NMR and ^13^C-NMR were recorded in DMSO on a Varian INOVA 500 NB Spectrometer using TMS as internal standard. Mass spectra were obtained on a Thermo LCQ DECA XP Spectrometer using ESI technique and Double Focussing Mass Spectrometer using FAB technique.

### 3.2. Synthesis of 5-fluorouracil-1-carboxylic acid (**3**)

Into a solution of 5-fluorouracil (6.5 g, 50 mmol) and potassium hydroxide (5.6 g, 100 mmol) in water (20 mL) was added a solution of chloroacetic acid (7.16 g, 75 mmol) in water (15 mL) and the resulting mixture was stirred at room temperature for 30 min. The pH value of the reaction mixture was adjusted to, and kept at 10 by the dropwise addition of a 10% aqueous potassium hydroxide solution. The mixture was then refluxed for 2 h, cooled, and acidified to pH 5.5 by the addition of concentrated hydrochloric acid, cooled at 4°C for 2 h, the crystals formed were isolated by suction and the solution set aside for further treatment. The solution was acidified to pH 2 by the addition of concentrated hydrochloric acid and cooled at 4°C for 2 h. The crystals were isolated by suction. Recrystallization was completed by dissolving the product in saturated aqueous sodium bicarbonate and reprecipitating with concentrated hydrochloric acid to produce white needles. Spectral data for **3 **were identical to those reported by Tada [27].

### 3.3. Synthesis of 5,7-dihydroxy-4-oxo-2-(3,4,5-trihydroxyphenyl)chroman-3-yl 2-(5-fluoro-2,4-dioxo-3,4-dihydropyrimidin-1(2H)-yl)acetate (**5**) and 2-(4-(2-(5-fluoro-2,4-dioxo-3,4-dihydropyrimidin-1(2H)-yl)acetoxy)-3,5-dihydroxyphenyl)-5,7-dihydroxy-4-oxochroman-3-yl-2-(5-fluoro-2,4-dioxo-3,4-dihydropyrimidin-1(2H)-yl)acetate (**6**)

A mixture of **1** (0.10 mmol), **3** (0.40 mmol), and *N ,N′*-dicyclohexylcarbodiimide (0.45 mmol), was stirred in THF (10 mL) for 6 h at room temperature. The reaction mixture was filtered and the filtrate was evaporated. The residue was separated by flash column chromatography (gradient elution with mixtures of dichloromethane-acetone) on silica gel and monitored by TLC. We collected and combined the second and the third bands, which contained the two products. The two collections were evaporated at room temperature and recrystallized to afford **5 **and **6**, respectively. Synthesized compounds **5 **and **6 ** were characterized by their melting points, ^1^H-NMR, ^13^C-NMR, IR, and MS analyses.

*Compound ***5***.* Yellow solid;62.3% yield; m.p. 282–283 °C. IR (KBr) ν_max _(cm^−1^): 3,295 (OH), 3,112 (NH), 3,015 (C=C), 1,756 (C=O), 1,643 (C=O), 1,458 (NH); ^1^H-NMR (DMSO) δ (ppm): 4.424 (dd, *J *= 17.7Hz, 2H, CH_2_), 5.378 (d, *J* = 11.1 Hz,1H, 2-H), 5.856 (d, *J *= 11.1 Hz, 1H, 3-H), 5.921 (d, *J *= 2.1 Hz, 1H, 8-H), 5.951 (d, *J *= 2.1 Hz, 1H, 6-H), 6.419 (s, 2H, 2’-H+ 6’-H), 7.976 (d, *J *= 7.1 Hz, 1H, 5-FU-6-H), 8.328 (s, 1H, 4’-OH), 9.004 (s, 2H, 3’-OH+ 5’-OH), 11.023 (s, 1H, 7-OH), 11.356 (s, 1H, 5-OH), 11.950 (s, 1H, 5-FU-3-NH); ^13^C-NMR (DMSO) δ (ppm): 48.01 (CH_2_), 73.09 (CH), 80.10 (CH), 95.37 (CH), 96.39 (CH), 100.61 (C), 106.66 (2CH), 125.03 (C), 130.08 (CH), 134.01 (C-OH), 140.32 (CF), 145.92 (2C-OH), 149.36 (C=O), 157.28 (C=O), 162.28 (C-O), 163.22 (C-OH), 166.88 (C=O), 167.43 (C-OH), 190.12 (C=O); ESI-MS m/z (%): 513.1 [M+Na]^+^; Anal. calcd. for C_21_H_15_FN_2_O_11_·3H_2_O(544.39): C, 46.33; H, 3.89; N, 5.15. Found: C, 46.17; H, 3.942; N, 5.246. 

*Compound* **6***.* Yellow solid; 28.6% yield; m.p. 291–293 °C IR (KBr) ν_max _(cm^−1^): 3,218 (OH), 3,078 (NH), 1,774 (C=O), 1,638 (C=O), 1,472 (N-H); ^1^H-NMR (DMSO) δ (ppm): 4.4609 (dd, *J *= 17.72 Hz, 2H, CH_2_), 4.8118 (s, 2H, CH_2_), 5.5219 (d, *J *= 4.88 Hz, 1H, CH), 5.8913 (d, *J *= 4.08 Hz, 1H, CH), 5.9588 (s, 2H, CH), 6.5256 (s, 2H, 2CH), 7.9248 (d, *J *= 18.24 Hz, 2H, 2CH), 9.7618 (s, 2H, 2OH), 11.3337 (s, 1H, OH), 11.9410 (s, 2H, NH); ^13^C-NMR (DMSO) δ (ppm): 48.03 (CH_2_), 48.08 (CH_2_), 72.92 (CH), 79.43 (CH), 95.49 (CH), 96.56 (CH), 100.37 (C), 106.48 (2CH), 126.72 (C), 129.91 (CH), 130.21 (CH), 133.38 (C-O), 138.30 (CF), 140.59 (CF), 145.59 (2C-OH), 149.50 (C=O), 149.97 (C=O), 157.23 (C=O), 157.48 (C=O), 162.03 (C-O), 163.20 (C-OH), 165.97 (C=O), 166.89 (C=O), 167.64 (C-OH), 189.61 (C=O); FAB-MS m/z (%): 661[M+H]^+^; Anal. calcd. for C_27_H_18_F_2_N_4_O_14_·4H_2_O(732.51): C, 44.27; H, 3.58; N, 7.65. Found: C, 44.46; H, 3.643; N, 7.525. 

### 3.4. Biological Evaluation

Cell growth inhibition assay: The K562 cell line was obtained from Sun Yat-sen Memorial Hospital, Guangzhou, China and the K562/ADR tumor cell line was obtained from the Institute of Hematology, Chinese Academy of Medical Science, Peking Union Medical College, Tianjin, China. The cells were cultured in a humidified atmosphere (37 °C 5% CO_2_) in RPMI 1640 medium supplemented with 10% FCS. According to the MTT method described in the literature [28,29], 1 × 10^4^ cells per well were seeded in 96-well plates and each group has four wells. Each well was respectively cultured for 24, 48 and 72 h with liquid medicine 100 μL that the concentrations of ADR ranged from 1.95 μmol/L to 250 μmol/L while concentrations of the other groups ranged from 0.625 μmol/L to 80 μmol/L as serial 1:2 dilutions. Then MTT solution (diluted in PBS, 5 mg/mL, 20 µL) was added to each well and incubated for 4 h. The formazan product was dissolved by adding DMSO (200 μL) to each well after centrifuged for 10 min under 2,000 rpm, and the absorbance value was measured at 570 nm. All measurements were performed in triplicate and each experiment was repeated six times. 

## 4. Conclusions

In summary, this is the first report on the synthesis and characterization of 5-Fu-substituted ampelopsin derivatives. The structures of two new derivatives were characterized by ^1^H-NMR, ^13^C-NMR, IR and MS, and their effects to inhibit the growth of K562 and K562/ADR cells were confirmed *in vitro*. This research will be helpful to develop novel anti-cancer agents based on combination principles.

## Supplementary Materials

Supplementary File 1
