# Supplementary material for: Synthesis and Biological Evaluation of New 5-Fluorouracil-Substituted Ampelopsin Derivatives
Source: Molecules. 2010 Mar 24;15(4):2114–23. doi: 10.3390/molecules15042114 (PMC6257404; doi:10.3390/molecules15042114)

Fig S1 Thermal gravimetric analysis of 5

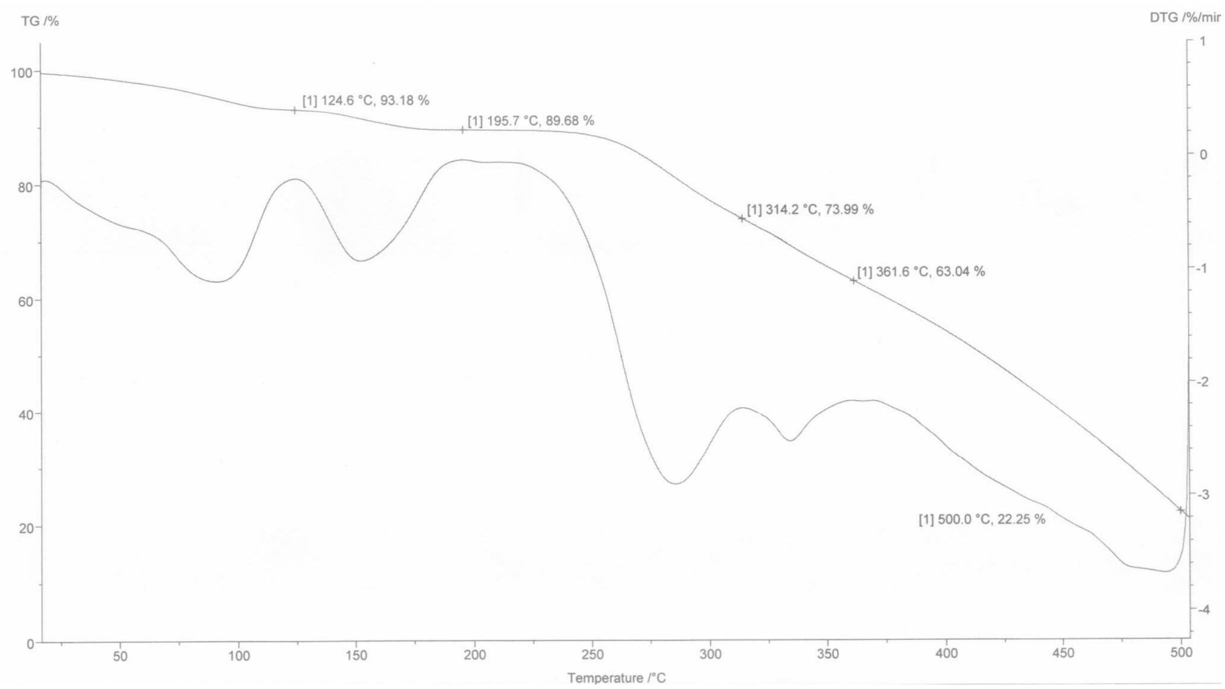

Fig S2 MS of 5

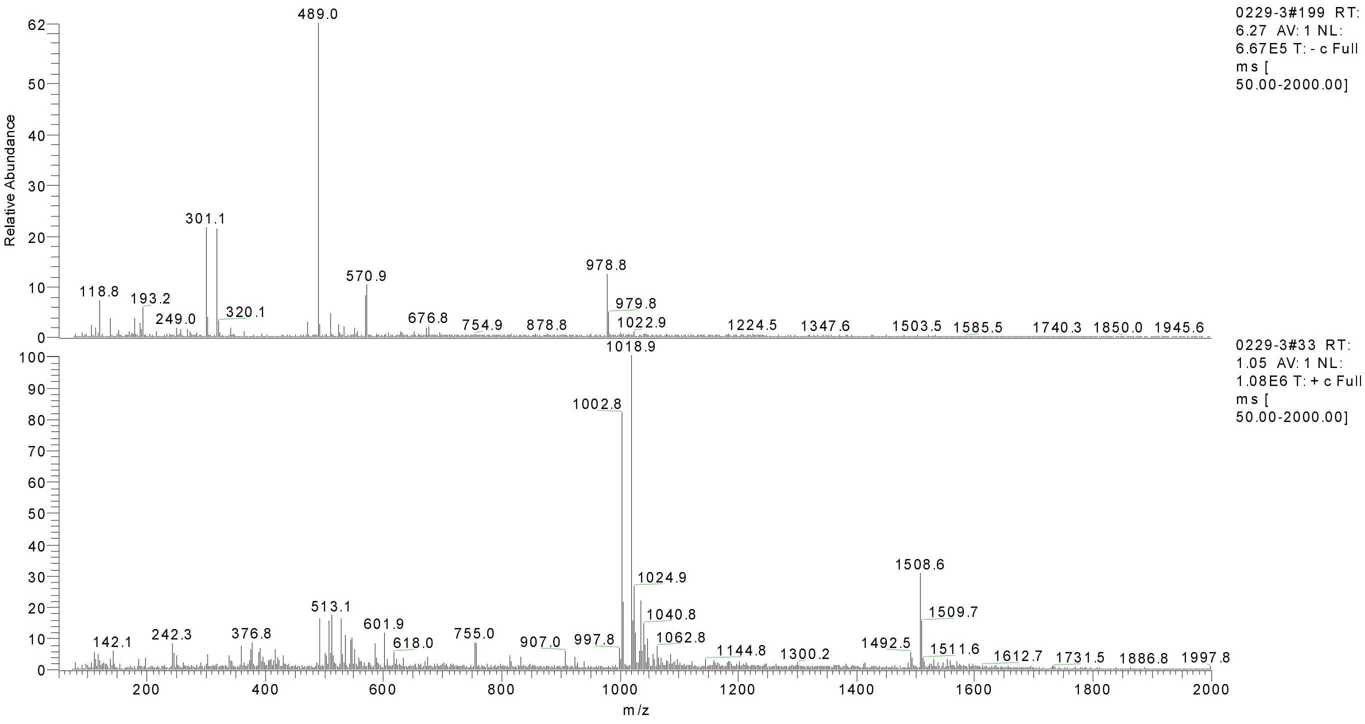

Fig S3 IR of 5

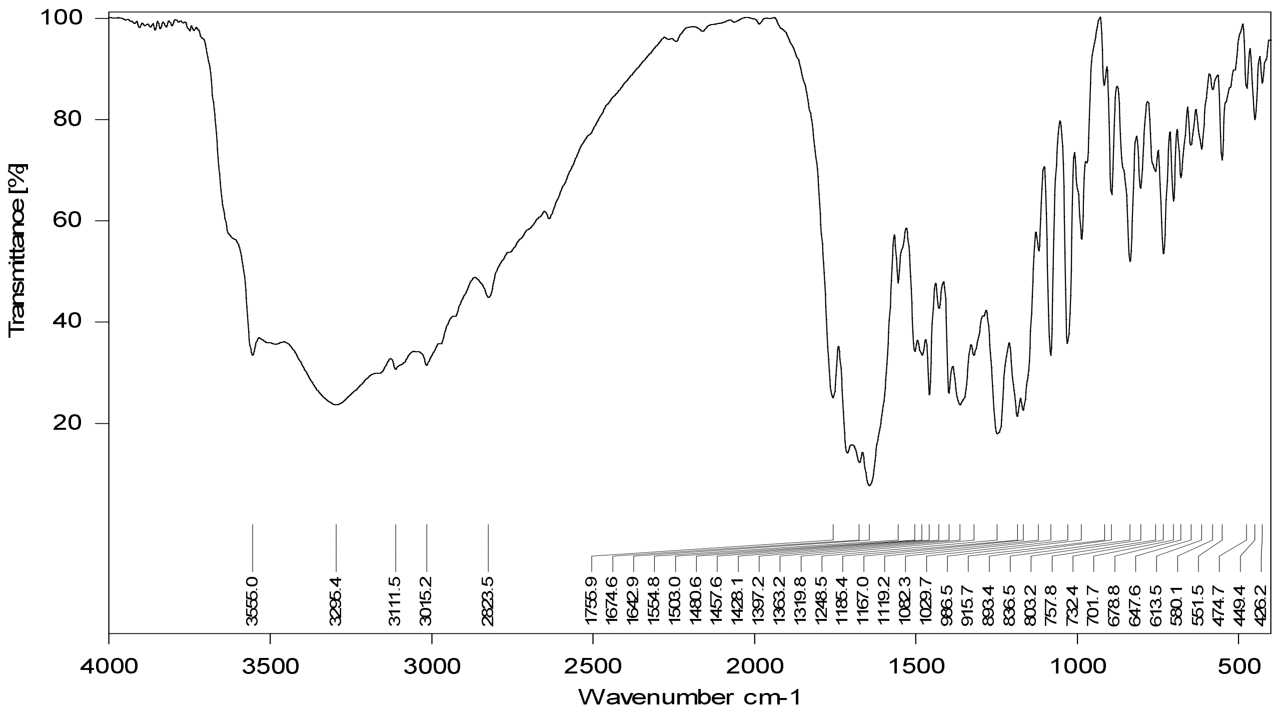

Fig S4 gCOSY spectrum of 5

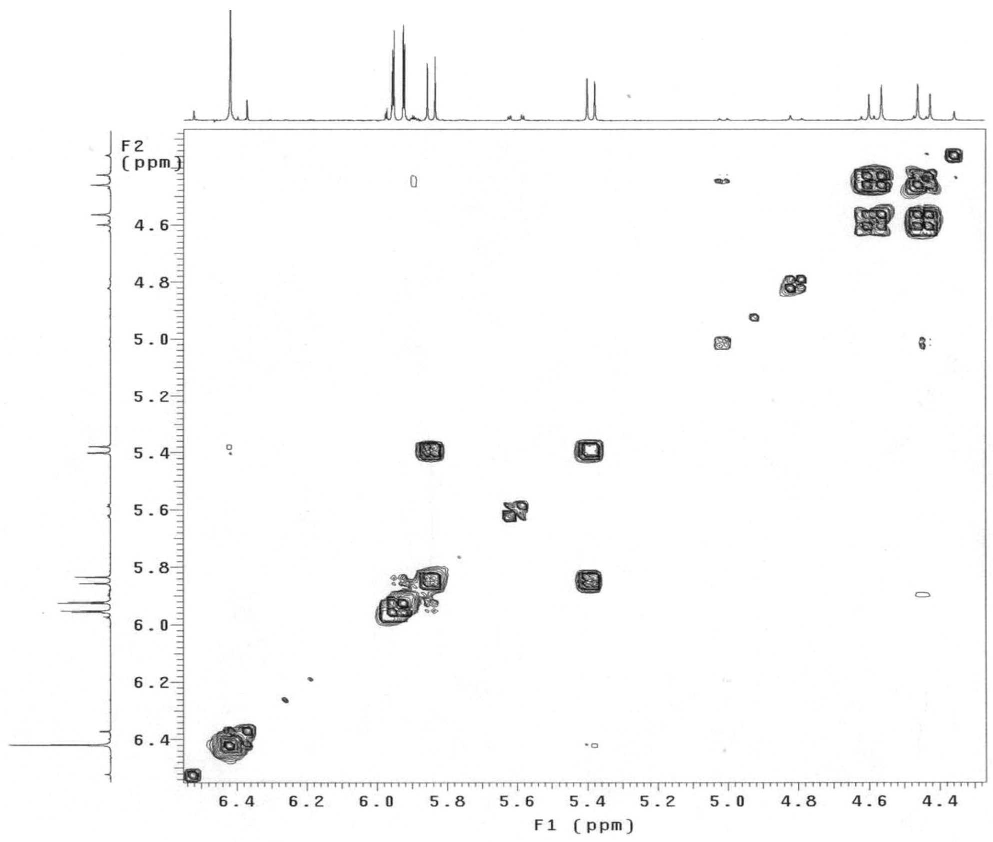

Fig S5 gHMQC spectrum of 5

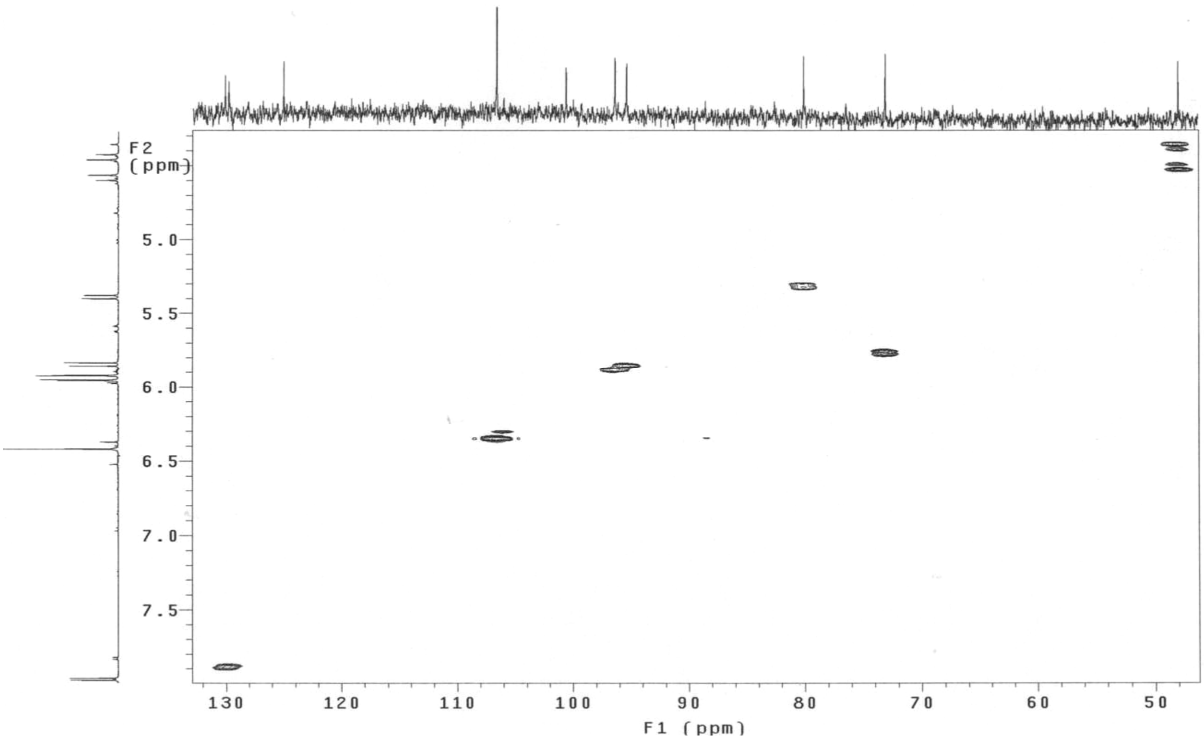

Fig S6 MS of 6

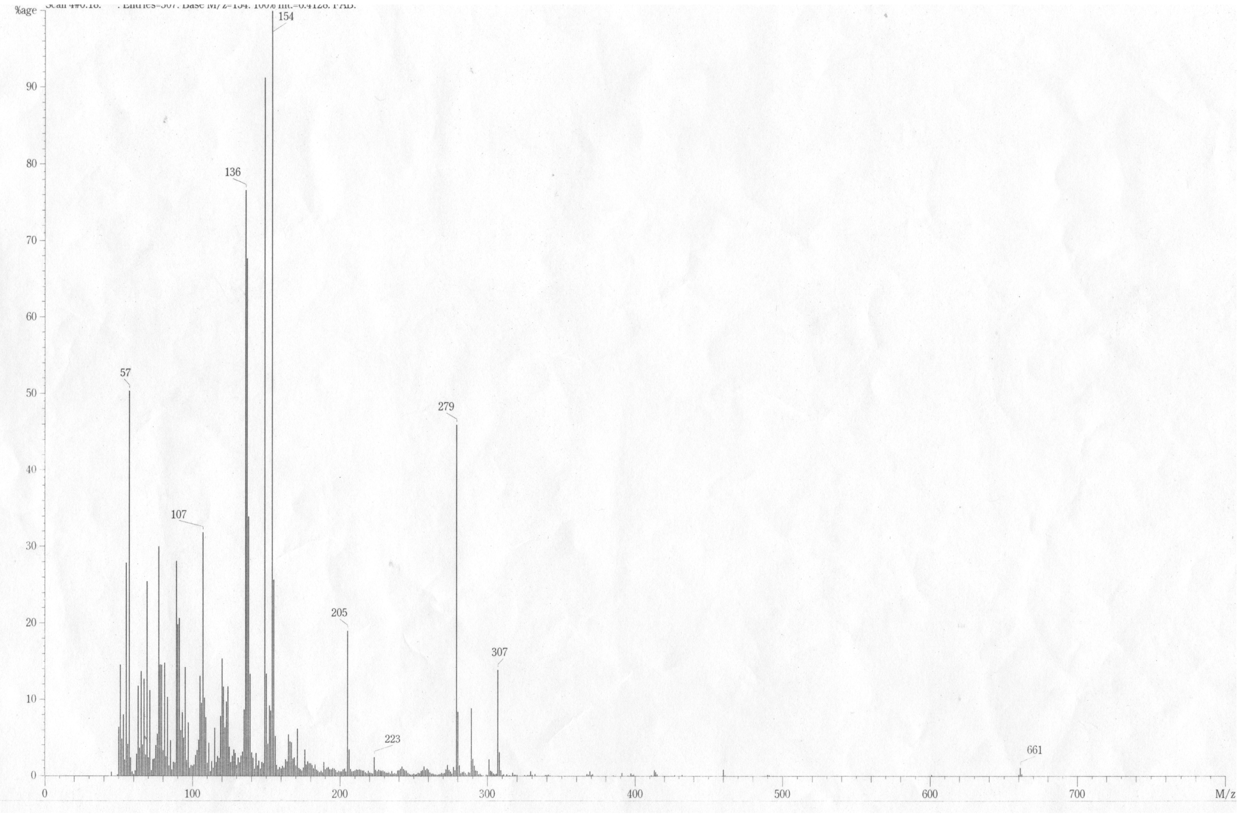

Fig S7 Thermal gravimetric analysis of 6

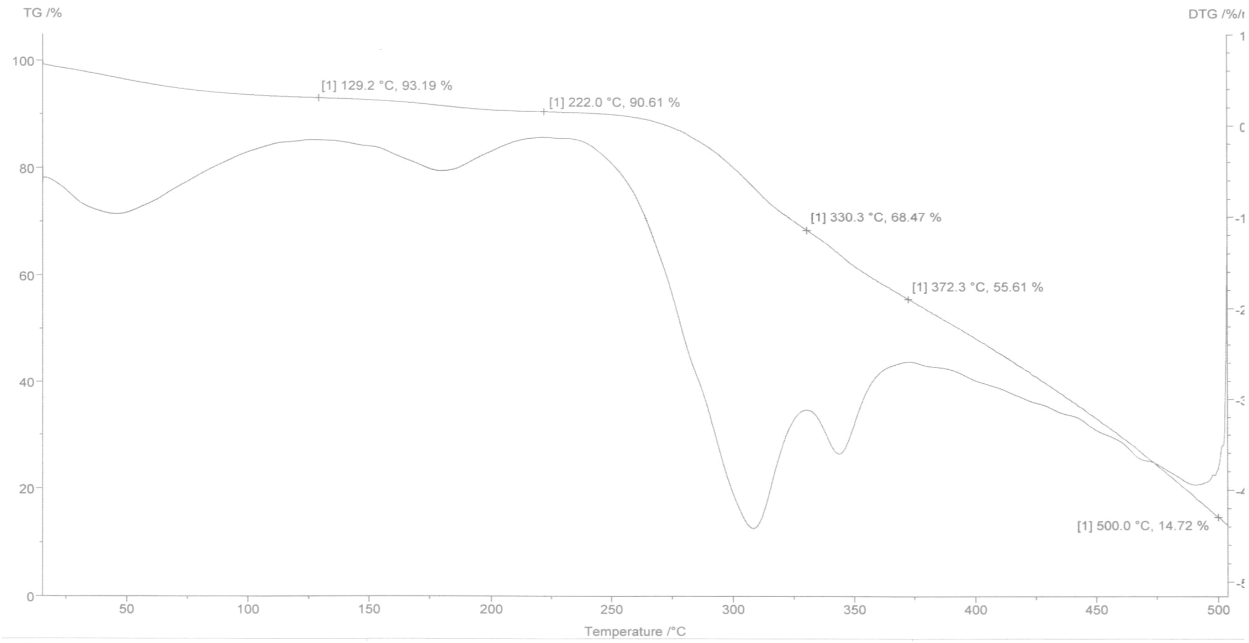

Fig S8 IR of 6

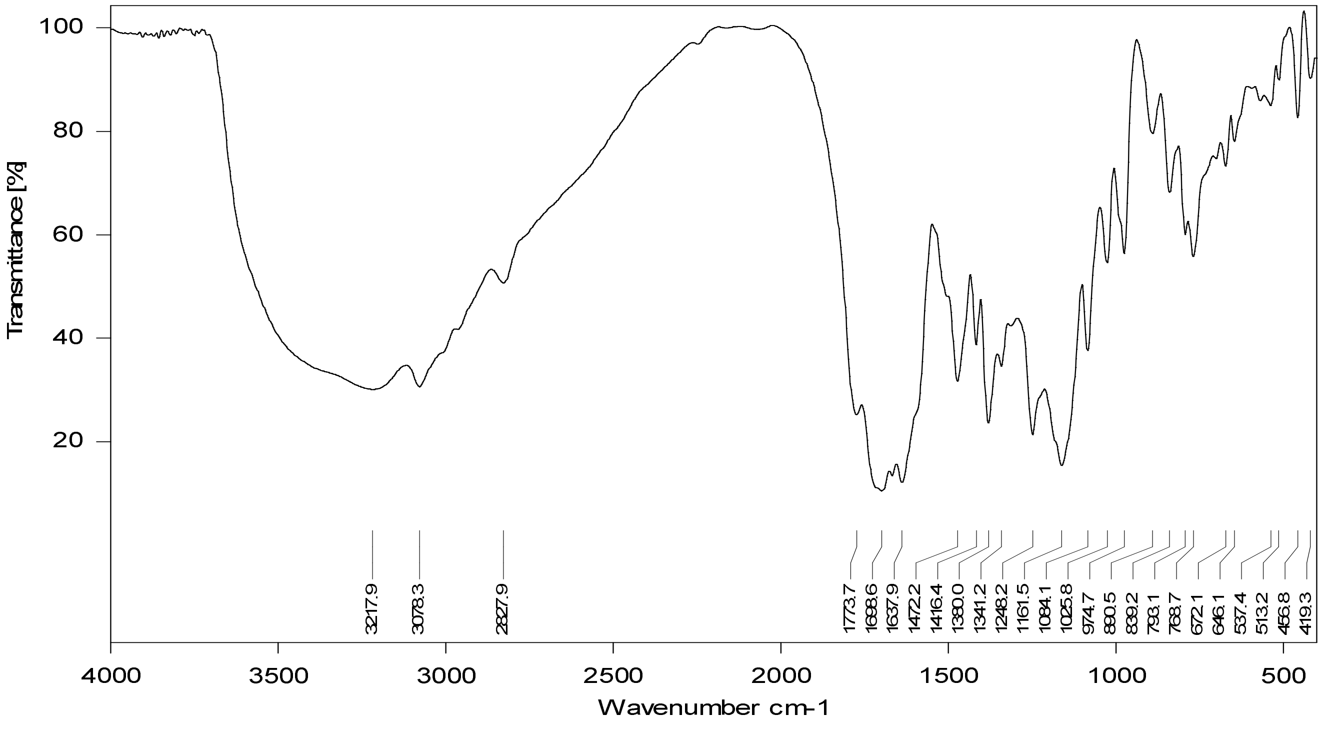

Fig S9 The inhibitory effects of **5** and **6** on K562 cells.

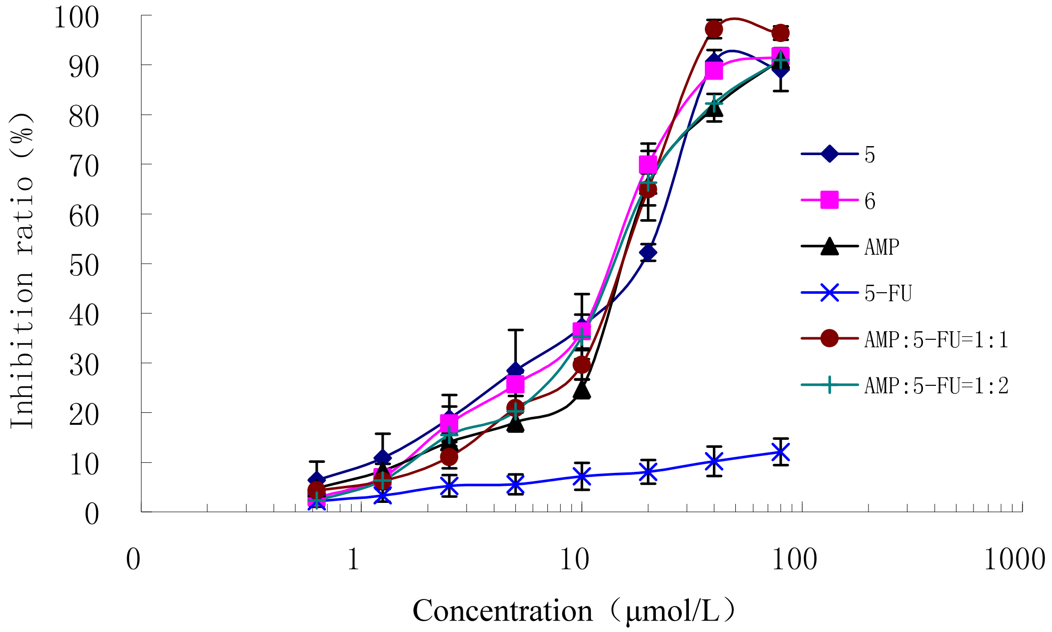

Fig S10 The inhibitory effects of **5** and **6** on K562/ADR cells.

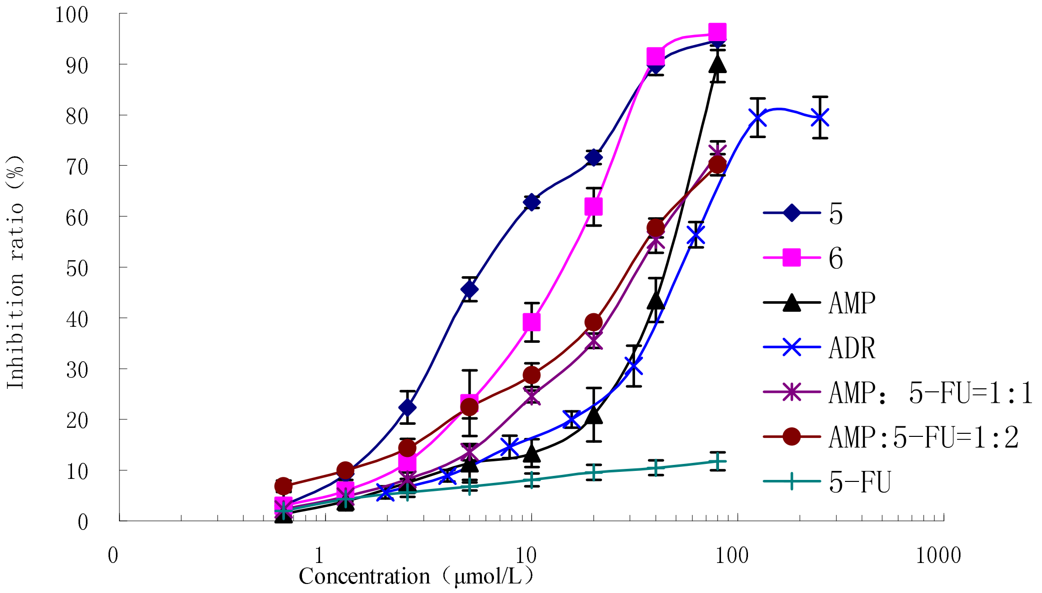

Fig S11 Reversal effect of **5** and **6** (C = 1.25 μmol/L) on multidrug resistance in K562/ADR cells for 48 h.

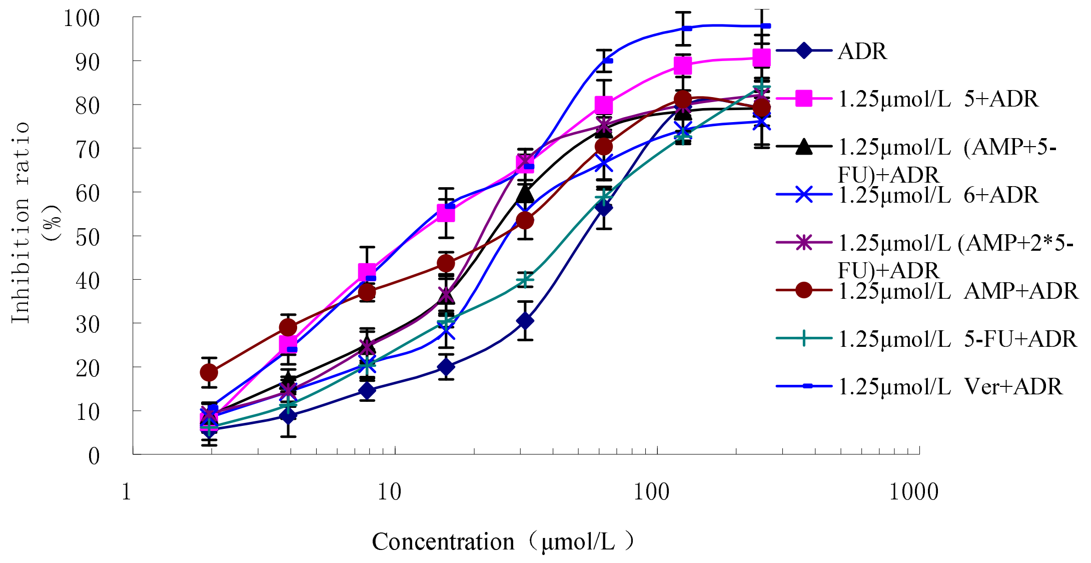

Fig S12 Reversal effect of **5** and **6** (C = 0.625 μmol/L) on multidrug resistance in K562/ADR cells for 48 h.

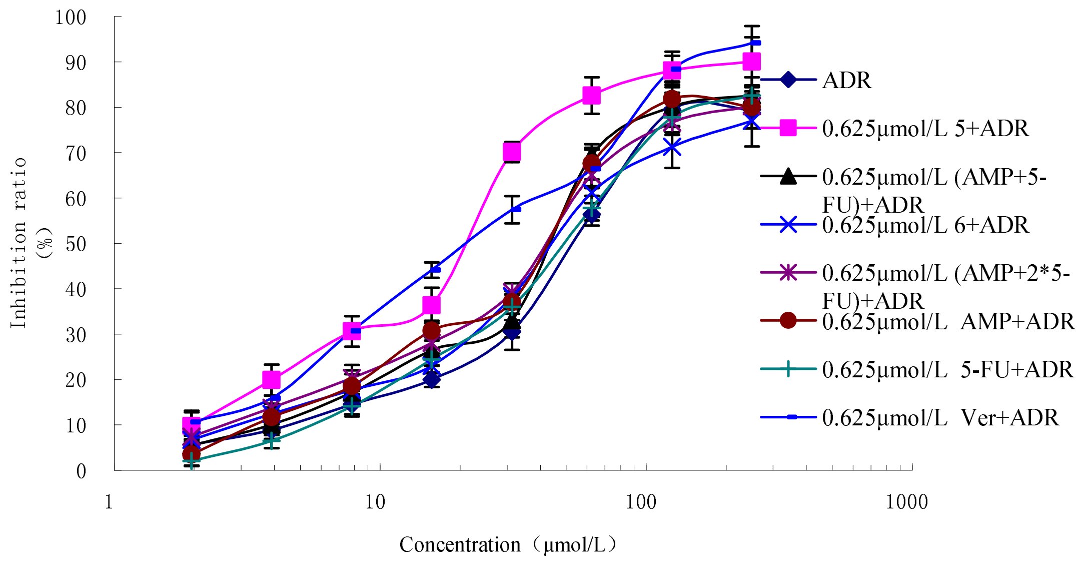

Supplement: Supplementary File 1 [file molecules-15-02114-s001.pdf]
